# Fabrication of Flexible Electrode with Sub-Tenth Micron Thickness Using Heat-Induced Peelable Pressure-Sensitive Adhesive Containing Amide Groups

**DOI:** 10.3390/nano11051250

**Published:** 2021-05-10

**Authors:** Hyebeom Shin, Eunseong Yang, Yong-Hoon Kim, Min-Gi Kwak, Youngmin Kim

**Affiliations:** 1 Display Research Center, Korea Electronics Technology Institute, 25 Saenariro, Bundang-gu, Seongnam 13509, Korea; hyebeom13@gmail.com (H.S.); didguswjd567@naver.com (E.Y.); kwakmg@keti.re.kr (M.-G.K.); 2 School of Advanced Materials Science and Engineering, Sungkyunkwan University, Suwon 16419, Korea; yhkim76@skku.edu; 3 SKKU Advanced Institute of Nanotechnology (SAINT), Sungkyunkwan University, Suwon 16419, Korea

**Keywords:** flexible electrode, sub-tenth micron thickness, heat-induced peelable, pressure-sensitive adhesive

## Abstract

In response to the increasing demand for flexible devices, there is increasing effort to manufacture flexible electrodes. However, the difficulty of handling a thin film is an obstacle to the production of flexible electrodes. In this study, a heat-induced peelable pressure-sensitive adhesive (h-PSA) was fabricated and used to manufacture a flexible electrode with sub-tenth micron thickness. Unlike the control PSA, the incorporation of amide groups made the h-PSA fail through adhesive failure at temperatures ranging from 20 to 80 °C. Compared to the peeling adhesion (1719 gf/in) of h-PSA measured at 20 °C, the value (171 gf/in) measured at 80 °C was decreased by one order of magnitude. Next, the 8 μm thick polyethylene terephthalate (PET) film was attached on a thick substrate (50 μm) via h-PSA, and Mo/Al/Mol patterns were fabricated on the PET film through sputtering, photolithography, and wet-etching processes. The thick substrate alleviated the difficulty of handling the thin PET film during the electrode fabrication process. Thanks to the low peel force and clean separation of the h-PSA at 80 °C, the flexible electrode of metal patterns on the PET (8 μm) film was isolated from the substrate with little change (<1%) in electrical conductivity. Finally, the mechanical durability of the flexible electrode was evaluated by a U-shape folding test, and no cracking or delamination was observed after 10,000 test cycles.

## 1. Introduction

Owing to its viscoelastic properties, a pressure-sensitive adhesive (PSA) wets an adherend within a short contact time and deforms to dissipate the applied energy during peeling [1,2,3,4]. This enables the PSA to possess high peel strength despite the joint being formed by only a slight pressure. Acrylate-based polymers are widely employed as PSA components because their adhesive properties are easily adjusted by copolymerizing various acrylic monomers [5]. For example, acrylates with low Tg improve the tack property and those with high Tg increase the cohesive strength [6]. However, the lack of strong intermolecular interactions causes PSA to fail through cohesive failure at high temperatures; thus, remnants of the PSA remain on the adherend. Given that an amide group can be engaged in hydrogen bonding [7], the cohesion force of a PSA is expected to be enhanced by incorporating amide groups in the acrylic polymer. In this study, we synthesized methacryloyl amide (Me-am 2) and used it to produce an acrylate polymer (Ac-am 3) containing amide groups. The structure of Me-am 2 was characterized by FTIR and NMR spectroscopy. Unlike the control PSA, the peel strength of the fabricated h-PSA using Ac-am 3 was high, and it failed adhesively because of intermolecular interactions. The amide hydrogen bonding allowed the h-PSA to be cleanly removed from the adherend via adhesive failure even at 80 °C. The viscoelastic properties of the h-PSA were analyzed by measuring the temperature dependence of the storage modulus and tan δ using a rheometer.

Flexible electrodes (e.g., graphene composites [8,9], silver composites [10,11,12,13,14], and ionic conductors [15]), which are light, thin, and shapeable, have received much attention as main components for the production of next-generation displays. Given that the thicker the electrode, the more is the applied stress on the electrode, the use of thin films for electrode fabrication is beneficial for securing its mechanical durability [16]. However, the handling of thin films during fabrication processes is challenging because of their poor mechanical property; therefore, thin films should be attached on a thick substrate using PSAs during these processes and then removed from the substrate by peeling when necessary. If the peeling adhesion of the PSA is high, the thin film is torn or wrinkled during peeling, and if the peel force of the PSA is low, the thin film is detached from the substrate during the manufacturing process. Hence, the PSA must have a high peel force to hold the thin film tight during the process and a low peel strength so that the thin film can be released easily by peeling. Although UV-curable PSAs have been used to manufacture flexible electrodes [16], the potential curing of these PSAs by photolithography limits their use [10]. In this study, a heat-induced peelable PSA (h-PSA) was utilized to fabricate a flexible electrode in which metal patterns were formed on an 8 μm polyethylene terephthalate (PET) film. Thanks to the combination of low peel force and adhesive failure of the h-PSA at 80 °C, the flexible electrode was cleanly detached from the substrate with a slight change (<1%) in its electrical properties to produce a sub-tenth micron thick electrode. The mechanical durability of the electrode was evaluated through a U-shape folding test.

## 2. Materials and Methods

### 2.1. Materials

Butyl acrylate, 2-ethylhexyl acrylate, ethyl acrylate, 2-hydroxyethyl acrylate, and triethylamine were purchased from Tokyo Chemical Industry (Tokyo, Japan). Dichloromethane, sodium bicarbonate, magnesium sulfate, celite, and methacryloyl chloride were purchased from Sigma-Aldrich Korea Ltd. (Yongin, Korea). Toluene and ethyl acetate were purchased from Samchun Chemical (Pyeongtaek, Korea), and 2,2-azobisisobutyronitrile (AIBN) was purchased from Junsei Chemical (Tokyo, Japan). All chemicals were used as received without further purification. A silicone-coated polyethylene terephthalate film and a polyethylene terephthalate film were purchased from SKC (Seoul, Korea). A stainless steel (SS) plate was purchased from MMSTECH (Bucheon, Korea).

### 2.2. Instrumentations

^1^H and ^13^C NMR spectra were measured on a nuclear magnetic resonance (NMR) spectrometer equipped with Bruker Top Spin 3.2 software (Ascend 500 MHz, Bruker, Madison, WI, USA). The Fourier transform infrared (FTIR) spectra in the range of 400 to 4000 cm^−1^ with a maximum resolution of 0.5 cm^−1^ were obtained by the attenuated total reflectance method using an FTIR spectrophotometer (IRAffinity-1S, Shimadzu, Kyoto, Japan). The glass transition temperature (Tg) and molecular weights were measured using a DSC-4000 (PerkinElmer, Waltham, MA, USA) with the heating rate of 10 °C/min and Agilent 1100 (Agilent, Santa Clara, CA, USA), respectively. The peel strength was measured on a tensile tester (HZ-1007E, MMSTECH, Bucheon, Korea). The viscosity was measured on a DV1 digital viscometer (AMETEK Brookfield, Middleboro, MA, USA). The temperature dependence of the shear storage modulus (G′) and dissipation factor (tan δ) was measured in the temperature range of 20 to 80 °C with a frequency of 1 Hz, a heating rate of 5 °C/min, and strain of 0.1% using a rheometer (MCR102, Anton Parr, Graz, Austria).

### 2.3. Synthesis of Methacryloyl Amide 2 (Me-am 2)

Hydroxyl amide 1 was prepared according to the reported method [17]. A three-necked flask was charged with hydroxyl amide 1 (5.0 g, 42.7 mmol), triethylamine (4.45 g, 44.0 mmol), and dichloromethane (50 g). To this solution methacryloyl chloride (4.46 g, 42.7 mmol) was slowly added at room temperature. After 20 h of stirring, methanol was added to the solution. Then, the mixture was poured into a separatory funnel and washed with an aqueous NaHCO_3_ solution, an aqueous HCl solution, and deionized water sequentially. An organic layer was collected in a flask, dried over MgSO_4_, and filtered through celite. The filtrate was concentrated under reduced pressure to afford a methacryloyl amide 2 as a viscous liquid.

^1^H NMR (400 MHz, CDCl_3_) δ: 6.12 (s, 1H), 6.09 (bs, 1H), 5.59 (s, 1H), 4.23 (t, 2H), 3.34 (q, 2H), 1.99 (s, 3H), 1.95 (s, 3H), 1.90 (quint, 2H).

^13^C NMR (100 MHz, CDCl_3_) δ: 170.5 (ester **C**=O), 167.5 (amide **C**=O), 136.2 (**C**=CH_2_), 125.7 (C=**C**H_2_), 62.3 (OH**C**H_2_), 36.4, 28.7, 23.1, 18.3.

### 2.4. Synthesis of Acrylic Polymer with Amide Functionality (Ac-am 3)

A three-necked flask (250 mL) equipped with a stirring bar, a condenser, and an N_2_-inlet was prepared. To the flask, the following materials were added: butyl acrylate (7.5 g), 2-ethylhexyl acrylate (4.5 g), ethyl acrylate (2.2 g), 2-hydroxyethy acrylate (0.62 g), methacryloyl amide 2 (0.78 g), AIBN (0.05 g), ethyl acetate (15 g), and toluene (3.76 g). The flask was immersed in an oil bath and heated at 80 °C. After 6 h of stirring, a viscous solution containing Ac-am 3 with a solid content of 45 wt% was obtained.

^1^H NMR (400 MHz, CDCl_3_) δ: 4.03 (bs, (CO)OC**H**_2_), 3.78 (s, C**H**_2_OH), 3.48 (s, (CO)NHC**H**_2_), 2.28 (s, (CO)C**H**), 1.98 (C**H**_3_NH(CO)), 1.90–0.88 (aliphatic region).

### 2.5. Synthesis of Ac-H

Acrylic polymer of Ac-H was synthesized through the previously described method using butyl acrylate (7.5 g), 2-ethylhexyl acrylate (4.5 g), ethyl acrylate (2.82 g), 2-hydroxyethy acrylate (0.78 g), AIBN (0.05 g), ethyl acetate (15 g), and toluene (3.76 g) as components. 

### 2.6. Peeling Adhesion Test

The as-prepared Ac-am 3 solution was bar-coated on a silicone-coated PET film (release liner), dried at 120 °C for 2 min, laminated onto a PET film, and kept at 80 °C for 16 h to produce an h-PSA tape with a multilayer structure of liner (50 μm)/PSA (20 μm)/PET film (50 μm). The h-PSA tape was cut into a 1 in wide strip. After the release liner was removed, the h-PSA tape was laminated onto an SS plate and kept at room temperature. After 30 min, the h-PSA/SS plate was placed on a tensile tester, and the h-PSA tape was subjected to a 180° peel test with a peeling rate of 300 mm/min at temperatures of 20, 40, 60, and 80 °C.

### 2.7. Lap Shear Test

A lap shear test was performed using a tensile tester [18]. For this, the 20 μm thick h-PSA was cut to form a 25 mm × 25 mm piece. Two SS plates measuring 100 mm × 25 mm were attached with an overlap area of 25 mm × 25 mm using the h-PSA. Both the ends of the SS plates were fixed in the grips and pulled in the tensile direction with a pulling rate of 0.5 mm/s at temperatures of 20, 40, 60, and 80 °C. The shear strain rate was calculated as

Shear strain rate (%) = ΔL/t × 100(1)

where ΔL is the displacement of the h-PSA, and t is the thickness.

### 2.8. Fabrication of Flexible Electrode 

**Figure 1:**
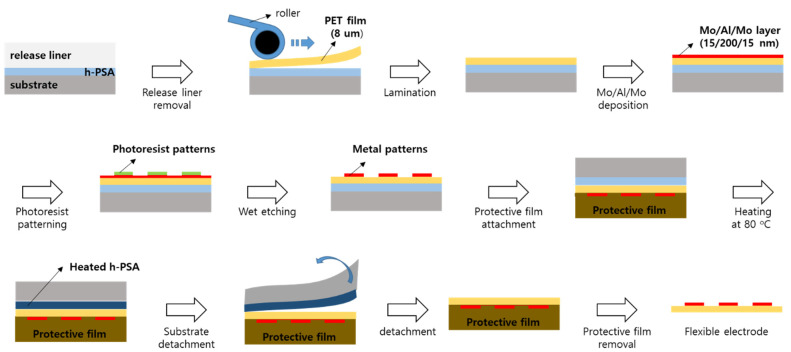
Schematic of the fabrication of the flexible electrode.

A schematic of the fabrication of the flexible electrode is shown in Figure 1. After the liner was removed, the h-PSA tape (7 cm × 7 cm) was laminated onto an 8 μm thick PET film to afford the structure of PET (8 μm)/h-PSA/substrate. This structure was placed in a DC sputtering system, and layers of molybdenum (15 nm), aluminum (200 nm), and molybdenum (15 nm) were sequentially deposited on the PET film in an argon atmosphere. The molybdenum and aluminum were deposited with DC powers of 150 and 300 W, respectively, under a working pressure of 4.6 × 10^−3^ Torr. Next, photoresist patterns were fabricated on the metal multilayer through photolithography, and the exposed metal layer was etched using an acidic solution. Then, the photoresist patterns were removed to produce Mo/Al/Mo patterns on the PET film. The structure of metal patterns/PET/h-PSA/substrate was laminated onto a protective film and then kept at 80 °C for 10 min. Subsequently, the protective film/metal patterns/PET structure was detached from the substrate. Thus, the flexible electrode of metal patterns/PET (8 μm) was isolated after the protective film was removed.

### 2.9. U-Shape Folding Test

**Figure 2:**
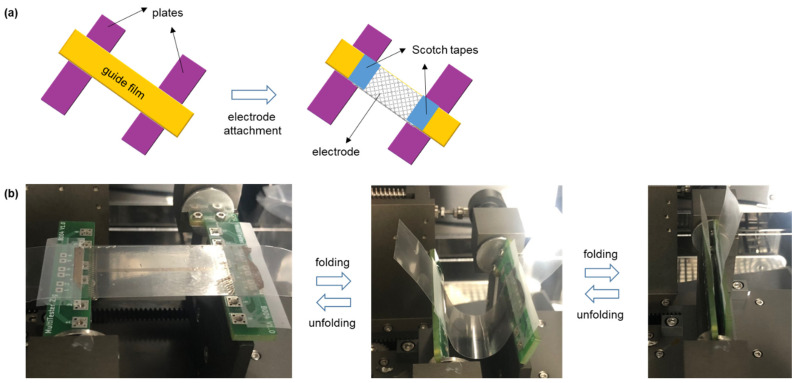
Schematics for (**a**) preparation of a U-folding test specimen and (**b**) folded and unfolded electrodes during a U-folding test.

Both ends of the as-prepared 4 cm × 6.5 cm electrode were attached on the guide film (50 μm), which was adhered to two plates using a Scotch tape (Figure 2a). After the plates were placed in a folding tester, the two plates were repeatedly moved towards each other to fold the electrode into a U shape and moved apart to unfold the electrode (Figure 2b). For this test, the folding radius, folding rate, and number of folding cycles were 2 mm, 25 cycles/min, and 10,000 cycles, respectively. 

## 3. Results

Because of the balanced cohesion force and free volume [19], PSAs are peeled from adherends with high peel strength via adhesive failure; thus, no debris of the PSA is left on the adherends [20]. However, the lack of strong intermolecular interactions generally causes cohesion failure when PSAs are peeled at high temperature [21]. To overcome this, a methacrylic monomer tethered with an amide group was synthesized and copolymerized with other acrylic monomers to produce a PSA in which intermolecular amide hydrogen bonding was expected.

**Scheme 1:**
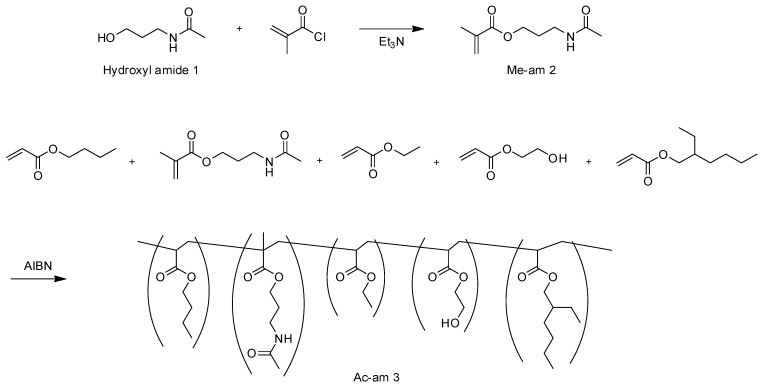
Synthesis of Me-am 2 and Ac-am 3.

**Figure 3:**
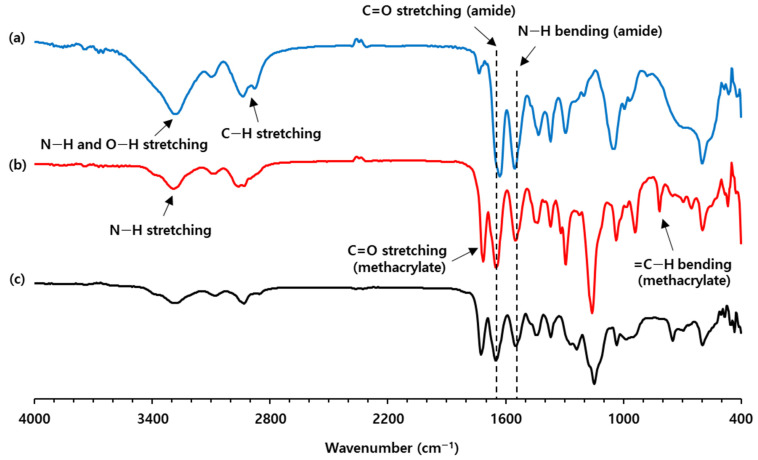
FTIR spectra of (**a**) hydroxyl amide 1, (**b**) Me-am 2, and (**c**) the UV-cured Me-am 2.

Firstly, methacryloyl amide (Me-am 2) was synthesized by reacting hydroxyl amide 1 with methacryloyl chloride at room temperature (Scheme 1). The novel compound 2 was characterized by FTIR and NMR spectroscopy. In the FTIR spectrum of Me-am 2, IR absorption for C=O stretching of the methacrylate group was observed at 1716 cm^−1^ (Figure 3) [22]. In addition, the presence of an amide bond in Me-am 2 was ascertained by intense IR absorption at 1647 and 1547 cm^−1^ corresponding to C=O stretching and N–H bending, respectively. In comparison to compound 1, the amide I band of compound 2 was shifted to high frequency by 19 cm^−1^ (Figure 3a,b). This indicates that the amide group of compound 1 is engaged in stronger hydrogen bonding than that of Me-am 2 [23]. This is because the hydrogen atom in the hydroxyl group acts as an H-bond donor, and the oxygen atom in the amide group serves as an H-bond acceptor. The possibility of amide–hydroxyl hydrogen bonding has previously been investigated through experiments [23,24] and calculation [25,26]. In the ^1^H NMR spectrum of Me-am 2, proton peaks for the methacryloyl group were observed at 6.12 and 5.59 ppm, and a broad proton resonance for NH(CO) appeared at 6.09 ppm. The presence of methacryloyl and amide groups was further confirmed by carbon signals at 170.5 and 167.5 ppm in the ^13^C NMR spectrum of Me-am 2. The radical polymerization of Me-am 2 was investigated by irradiating the mixture of Me-am 2 and a photoinitiator (Irgacure-184) with UV light. In the FTIR spectrum of the cured Me-am 2, the intensity of the IR absorption at 813 cm^−1^ for =C–H bending decreased, confirming polymerization of the methacryloyl groups (Figure 3c). The thermal behavior of the cured Me-am 2 was observed in the temperature range of −70 to 200 °C by differential scanning calorimetry (DSC). Two endothermic peaks were observed at −10 and 154 °C, which were assigned as the glass transition temperature (Tg) and melting temperature of the cured Me-am 2, respectively.

With this compound in hand, the acrylic polymer (Ac-am 3) containing amide groups was synthesized (Scheme 1) and used to fabricate the h-PSA. To realize the tack property, acrylic monomers with chain lengths of C4 to C8 were used as the main components for synthesizing Ac-am 3. In addition, 2-hydroxyethyl acrylate (HEA) was also employed as a component for the acrylic polymer because strong hydrogen interactions between the hydroxyl groups and the amide groups were expected. Through free-radical polymerization, an acrylic polymer containing hydroxyl and amide groups was synthesized and isolated. In the ^1^H NMR spectrum of Ac-am 3, the proton resonances for C**H_2_**OH and NH(CO)C**H_2_** were observed at 3.78 and 3.48 ppm, respectively, confirming the incorporation of hydroxyl and amide groups in the acrylate backbone. The viscosity of the solution containing Ac-am 3 with a solid content of 45 wt% was measured as 4500 cps at 20 °C. The number average and weight average molecular weight of Ac-am 3 were measured to be 56 and 435 kg/mol, respectively, with a polydispersity index of 7.77. The Tg of Ac-am was −43 °C (obtained by DSC), and this value was close to that (−45 °C) calculated by the Fox equation.

**Figure 4:**
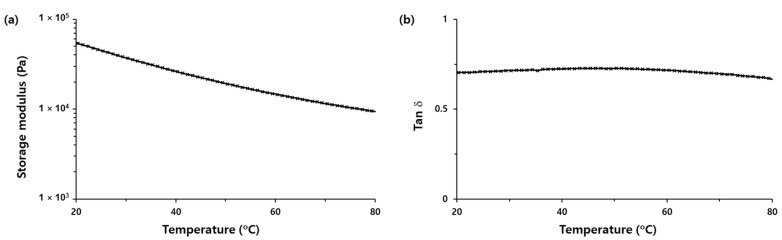
Temperature dependence of (**a**) storage modulus and (**b**) tan δ of h-PSA.

Next, the Ac-am 3 solution was bar-coated on a silicone-coated PET film (release film) and dried to produce a 20 μm thick h-PSA layer on the release film. Then, a PET film (50 μm) was laminated onto the h-PSA layer to produce an h-PSA tape with the structure of release film/h-PSA/PET film. The peeling adhesion of the h-PSA tape was measured as 1719 (±60) gf/in at 20 °C. Interestingly, no residue remained on the adherend after detachment, implying that the h-PSA failed adhesively. In comparison, we synthesized an acrylic polymer (Ac-H) without Me-am 2 and used it to fabricate a control PSA. The peel strength of the control PSA was measured as 583 (±60) gf/in, and the residual PSA was observed on the adherend after detachment. Given that the PSA without strong intermolecular interactions generally failed cohesively, the amide hydrogen bonding of Ac-am 3 enabled the h-PSA to be practically usable. Encouraged by this result, we measured the peel strength of the h-PSA tape by a 180° peel test at various temperatures (20, 40, 60, and 80 °C) and analyzed the failure modes. The peel strength of the h-PSA at 40, 60, and 80 °C were measured as 612 (±67), 363 (±10), and 171 (±24), respectively, and all h-PSAs failed adhesively. The temperature dependence of peeling adhesion is summarized in Table 1. In comparison to 20 °C, the peeling adhesion of the h-PSA tape was decreased by one order of magnitude at 80 °C due to the softening of the h-PSA. The softening of the h-PSA was confirmed by a decrease in the storage modulus (G′) with increasing temperature from 20 to 80 °C (Figure 4a). However, the dissipation factor (tan δ) was almost constant and less than 1 in this temperature range (Figure 4b). This indicates that the h-PSA behaved like a viscoelastic solid rather than a liquid even at 80 °C [27]; thus, the h-PSA failed adhesively. The combination of low peeling adhesion and clean separation made the h-PSA tape peelable at 80 °C.

**Figure 5:**
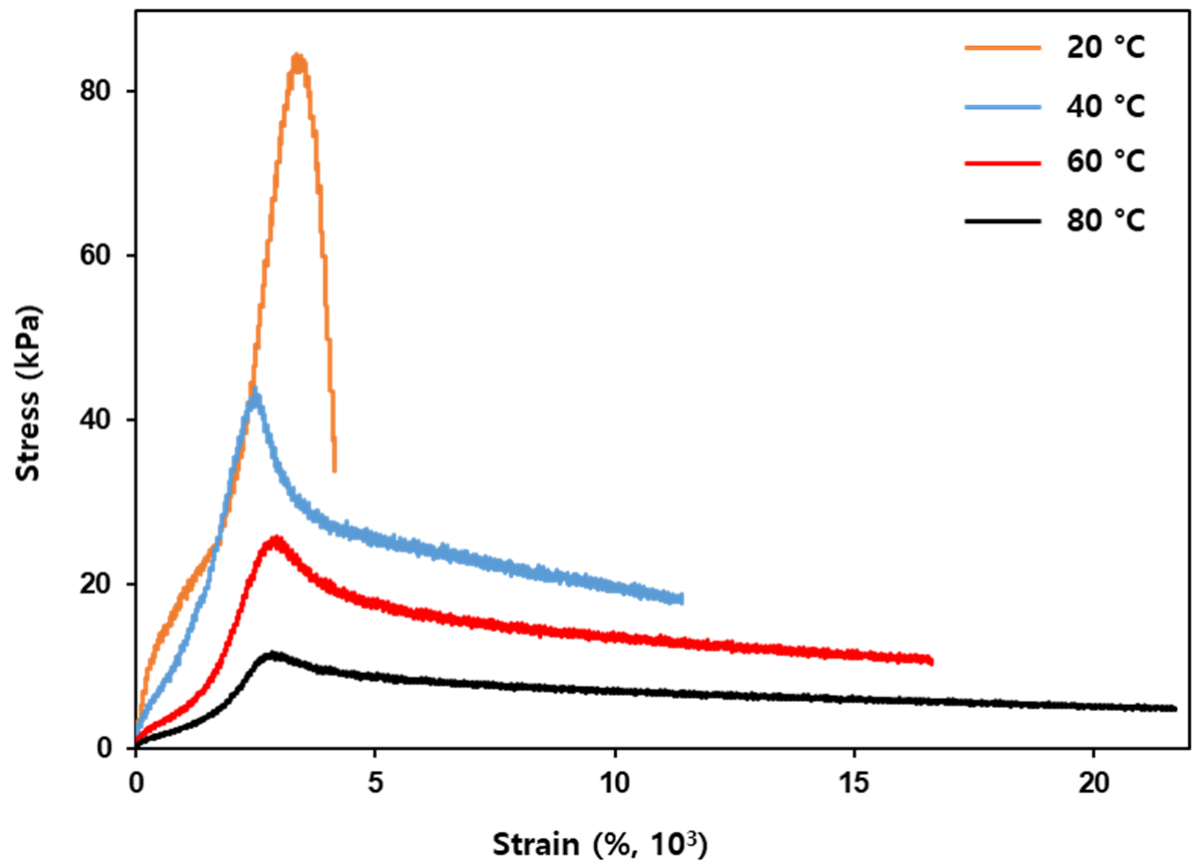
Temperature dependence of stress–strain curves of h-PSA.

The temperature dependence of the mechanical properties of the h-PSA was investigated (Figure 5), and the results are summarized in Table 1. As the temperature was increased from 20 to 80 °C, the maximum strength of the h-PSA decreased from 81.1 (±4.2) to 12.1 (±3.7) kPa, and the elongation at the breaking point increased from 5.6 (±1.3) × 10^3^% to 22.0 (±5.8) × 10^3^%. The change in mechanical properties with increasing temperature was in good agreement with the storage moduli obtained by the rheometer.

**Figure 6:**
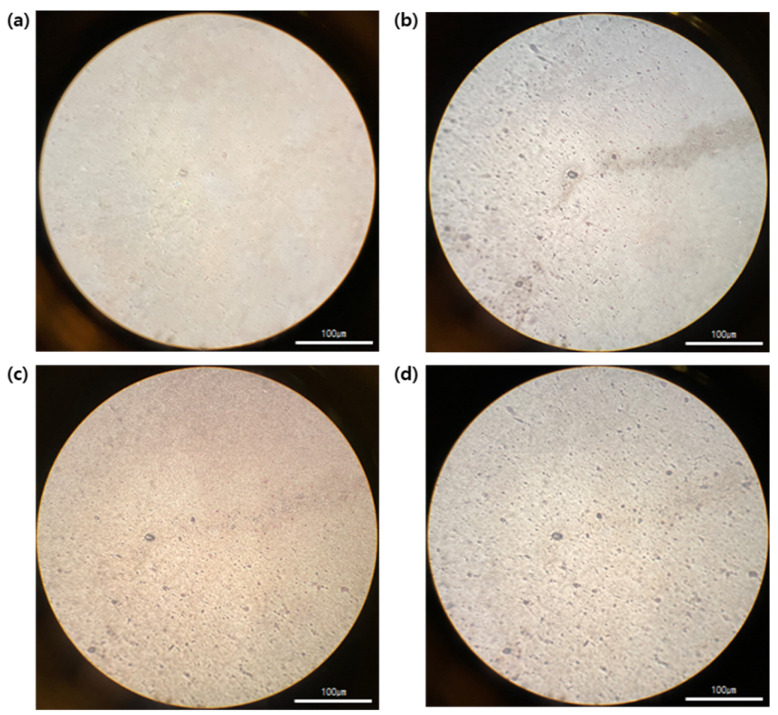
Microscopic images of the surfaces of the (**a**) PET film, (**b**) Mo layer in the Mo/PET film, (**c**) Al layer in the Al/Mo/PET film, and (**d**) Mo layer in the Mo/Al/Mo/PET film.

**Figure 7:**
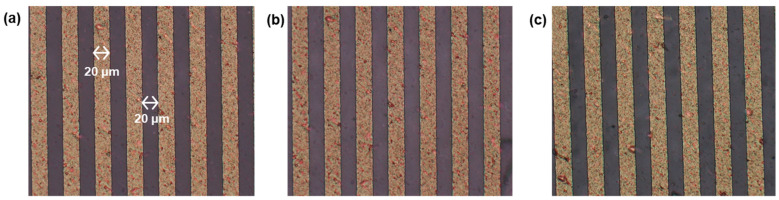
Microscopic images of the metal patterns on the 8 μm thick PET film (**a**) before and (**b**) after detachment. (**c**) A microscopic image of the metal patterns of the flexible electrode after 10,000 folding test cycles.

Next, a flexible electrode with sub-tenth micron thickness was fabricated using a PSA (Figure 1). Considering the difficulty of handling a thin film (<10 μm), it must be fixed on a thick substrate via a PSA during fabrication and separated from the substrate as a free-standing film by peeling. Therefore, the high peel strength of the PSA should be reduced when necessary, and no residue of the PSA should remain on the film after detachment. In this work, h-PSA was utilized to fabricate an 8 μm thick flexible electrode (Figure 1). The h-PSA tape was laminated onto an 8 μm thick PET film. Then, metal layers of Mo (15 nm), Al (200 nm), and Mo (15 nm) were sequentially deposited on the PET by DC sputtering. The surface resistance of the Mo/Al/Mo multilayer was measured as 0.37 (±0.03) Ω/sq. Figure 6 shows the microscopic photos of the surface of the pristine 8 μm thick PET film and each metal layer fabricated by sequential sputter deposition. The surface morphology of the metal layers was similar to that of the PET film because the metal layers were too thin (15–200 nm) to cover the roughened surface of the PET film. Next, metal patterns with a 20 μm line width were made on the PET film through photolithography and wet-etching processes (Figure 7). The line resistance of the patterns was determined as 8.89 (±0.40) × 10^2^ Ω. Then, the metal patterns on the PET film were laminated onto a protective film to prevent them from being scratched during the peeling process. The structure of protective film/patterned metal layer/PET(8 μm)/h-PSA/substrate was kept at 80 °C for 10 min during which the peel strength of the h-PSA was reduced; thus, the structure of the protective film/patterned metal layer/PET(8 μm) was readily detached from the substrate. After the protective film was removed, the flexible electrode of the patterned metal layer/PET(8 μm) was isolated as a free-standing film. The line resistance of the flexible electrode was slightly increased to 8.97 (±0.36) × 10^2^ Ω after detachment, indicating that reduction in the peel strength of the h-PSA at 80 °C played a key role in the fabrication of the thin electrode through a temporary bonding and debonding process. No cracking or delamination was observed on the surface of the flexible electrode by microscopy (Figure 7b), and this was in good agreement with the little change (<1%) in conductivity of the electrode after detachment.

The mechanical durability of the flexible electrode was evaluated through a U-shape folding test [22]. Both the ends of the electrode were attached on a guide film adhered to two plates using a Scotch tape (Figure 2a). During the folding cycles, the two plates were repeatedly moved towards each other to fold the electrode into a U-shape and moved away from each other to unfold the electrode (Figure 2b). The folding radius and folding rate for the test were 2 mm and 25 cycles/min, respectively. The line resistance of the flexible electrode was measured as 9.03 (±0.37) × 10^2^ Ω after 10,000 folding test cycles, implying that the electrode was mechanically durable. No cracking or delamination of the Mo/Al/Mo layers was observed by microscopy (Figure 7c) after numerous folding cycles with a narrow folding radius. This mechanical durability was ascribed to the pliability of the electrode with a sub-tenth micron thickness.

## 4. Conclusions

In this study, amide-tethered methacrylate (Me-am 2) was synthesized and used to fabricate an h-PSA. The amide hydrogen bonding not only endowed the h-PSA with high peel strength (1719 gf/in) but also made it fail adhesively at 20 °C. The peeling adhesion was decreased by one order of magnitude at 80 °C, and no adhesive residue was observed after detachment of the h-PSA. This heat-induced peelable PSA was used to fabricate a flexible electrode through a temporary bonding and deboning process. Thanks to the low peeling strength and clean separation of the h-PSA at 80 °C, the flexible electrode with metal patterns on an 8 μm thick PET film was isolated by peeling without damage. The sub-tenth micron thickness made the electrode mechanically durable, and it showed little change in its electrical properties after 10,000 U-shape folding test cycles. Given that electronics are becoming smaller, lighter, and more flexible, the demand for thinner electrodes is increasing. Therefore, the proposed temporary bonding and debonding process employing a heat-induced peelable PSA is expected to be a breakthrough that will enable the production of thin electrodes without damage.

## Figures and Tables

**Table 1:** The peel strength and mechanical properties of h-PSA at different temperatures.

Temperature (°C)	Peel Strength (gf/in)	Maximum Strength (kPa)	Elongation at Break (%, 10^3^)
20	1719 (±60)	81.1 (±4.2)	5.6 (±1.3)
40	612 (±67)	44.8 (±7.0)	12.0 (±0.8)
60	363 (±10)	24.4 (±1.8)	19.3 (±4.8)
80	171 (±24)	12.1 (±3.7)	22.0 (±5.8)

## Data Availability

Data sharing not applicable.
